# Serum IL-36β levels are associated with Insulin sensitivity in paediatric patients with obesity

**DOI:** 10.1038/s41366-024-01508-4

**Published:** 2024-03-11

**Authors:** Paloma Narros-Fernández, Andrew O’Donnell, Clodagh Sheehy, Shrikanth Chomanahalli Basavarajappa, Yasmina Esther Hernandez Santana, David Kinlen, Declan Cody, Andrew E. Hogan, Patrick T. Walsh

**Affiliations:** 1 Trinity Translational Medicine Institute, School of Medicine, Trinity College Dublin, Dublin, Ireland; 2 National Children’s Research Centre, CHI Crumlin, Dublin, 12 Ireland; 3 Departments of Paediatric Endocrinology, Children’s Health Ireland at Crumlin, Dublin, Ireland; 4 Kathleen Lonsdale Institute for Human Health Research, Maynooth University, Maynooth, County Kildare, Ireland

**Keywords:** Obesity, Obesity

## Abstract

Although the orchestrating role of Interleukin-36 cytokines in regulating inflammation at barrier tissue sites, is well established, whether they play a significant role in the settings of metabolic health and disease, has yet to be fully established. Several recent studies have demonstrated that IL-36 cytokine expression is elevated among adult patients with obesity, and can play roles in regulating both insulin sensitivity and driving inflammation. In this report, we have extended these analyses to paediatric patients and identified an association between elevated serum levels of expression of the specific Interleukin-36 subfamily member, IL-36β, among children with obesity displaying insulin sensitivity, compared to children with obesity who are insulin resistant. While these data further indicate a possible protective role for IL-36 in metabolic health, they also differ with previous findings from an adult patient cohort, where elevated levels of the related cytokine, IL-36γ, were found to occur in association with improved metabolic health. While highlighting important differences between paediatric and adult patient cohorts in the context of metabolic disease associated with obesity, these data underscore the need for a deeper mechanistic analysis of the role of IL-36 cytokines in disease.

Interleukin-36 (IL-36) cytokines form a subset of the larger IL-1 family of cytokines with emerging roles in health and disease across several tissue sites. The IL-36 subfamily consists of three cytokines designated IL-36α, β and γ and a specific IL-36 receptor antagonist IL-36Ra [1]. Members of the IL-36 subfamily mediate cellular responses through engagement with the IL-36 receptor complex, comprised of the IL-1RL2 and IL-1RAcP proteins. While the influence of these cytokines in regulating inflammatory responses at barrier sites, such as the skin and the gut, have been established, what influence they may play in regulating metabolic health and disease, particularly in the setting of obesity, has been relatively understudied [2–4]. Initial studies, by ourselves and others, have focused on adults with obesity (AWO), whom had established Type 2 diabetes (T2D), to determine whether IL-36 cytokines may play a role in disease. These studies all indicate that serum levels of IL-36 cytokines, specifically IL-36α and γ, are elevated in AWO and may be important mediators of adipose tissue and peripheral inflammation in the context of T2D [5–7]. Interestingly, we have also previously demonstrated that increased levels of IL-36γ were associated with lower blood glucose levels among AWO and co-morbid T2D, indicating that IL-36 cytokines can play a role in maintaining metabolic health. Furthermore, murine studies confirmed a protective role for the IL-36 family in the setting of obesity while indicating these effects were influenced by dysbiosis in the intestinal microbiome [5].

Increased rates of childhood obesity represents a significant health challenge with consequent metabolic dysfunction predisposing to a range of comorbidities later in life. As such, in an effort to extend previous observations, we have now examined whether peripheral levels of IL-36 cytokines are altered in children with obesity (CWO) during the earliest stages of metabolic disease onset. Alongside related IL-1 family members (IL-1β, IL-18 and IL-33), we measured serum levels of IL-36 family cytokines in a cohort of CWO (BMIz score >2)(*n* = 61) ranging in age from 7-18 years, comparing to a control age matched cohort of healthy bodyweight children (HC) (BMIz score<2) (*n* = 52) (see sTable 1 for patient details).

These analyses revealed several differences when compared to previous reports based upon adult patients. Most notably, levels of all IL-36 cytokines were similar between HC and CWO, indicating that, unlike in adult patients, serum levels of IL-36 are not altered in childhood obesity (Fig. s1A–D). In contrast, levels of IL-18 were found to be elevated in obesity as previously described (Fig. s1E) [8]. IL-1β and IL-33 were found to be low or not detected among all patients tested (data not shown). Next, CWO were segregated on the basis of insulin sensitivity or resistance using HOmeostatic Model Assessment for Insulin Resistance (HOMA-IR) model which predicts pancreatic-cell function and insulin sensitivity for a given combination of fasting levels of plasma glucose and insulin [9]. When CWO were separated on the basis of those who were Insulin resistant (HOMA-IR > 3), versus Insulin sensitive (HOMA-IR < 3), levels of the IL-36 family member, IL-36β were found to be significantly elevated among insulin sensitive patients (Fig. 1A). A deeper analysis of IL-36β levels among CWO demonstrated a significant negative correlation with HOMA-IR score (Spearman test, r = -0.33, p = 0.004) whereas no correlation was seen with obesity levels (BMIz score) (Fig. 1B, C). No other IL-36 family members were found to be significantly altered when patients were segregated on this basis (Fig. s1F–H). These data indicate that, similar to observations in adult cohorts, IL-36 cytokines are elevated among CWO with normal levels of Insulin sensitivity versus those displaying Insulin resistance. In contrast, and in agreement with previous reports, levels of IL-18 were found to be significantly increased among CWO displaying insulin resistance and were positively correlated with levels of obesity but not insulin sensitivity (Fig. 1D–F) [8].

**Fig. 1:**
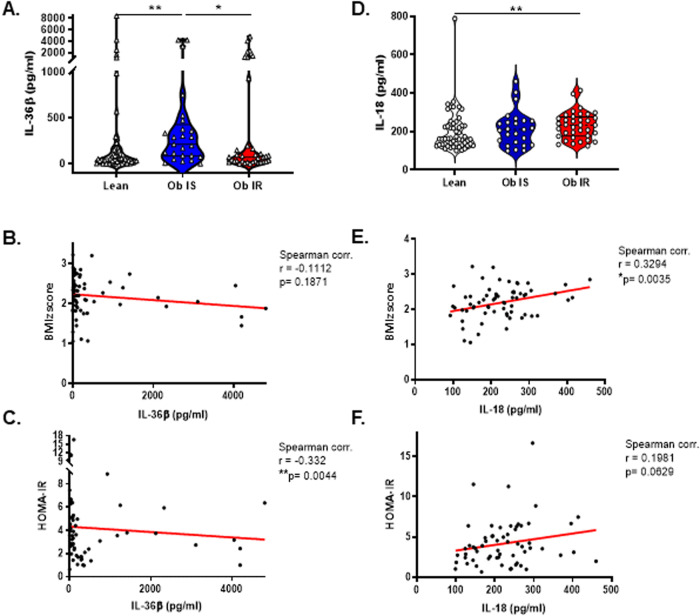
Serum levels of IL-36β and IL-18 cytokines in healthy bodyweight controls compared to both insulin-sensitive and insulin-resistant cohorts of children with obesity. Serum levels of (**A**) IL-36β, and (**D**) IL-18 were measured in patients serum by ELISA. Children with obesity were sub-classified as either insulin sensitive (IS) (*n* = 25) or insulin resistant (IR) (*n* = 36) based on HOMA-IR score. Correlation of serum IL-36β and IL-18 levels with BMIz scores (**B**, **E**) the homeostatic model assessment for insulin resistance (HOMA-IR) (**C, F**) among children with obesity (*n* = 61). Data in (**A**) and (**D**) are shown as means ± SEM. Statistical analysis by two-tailed Mann-Whitney test (*p ≤ 0.05, **p ≤ 0.01). For data in (**B**), (**E**), (**C**) & (**F**), Spearman correlation coefficients (r) and corresponding p values shown (*p ≤ 0.05, **p ≤ 0.01).

While these observations are further suggestive of a protective role for IL-36 family members in metabolic health and disease, they raise some interesting questions. For example, it is notable that the altered IL-36 family members associated with this protection differ between adult and childhood cohorts examined. In adult cohorts, it has been reported that IL-36γ is elevated in obesity and correlates with improved metabolic health, whereas in childhood cohorts, IL-36β is the family member associated with insulin sensitivity. While the reasons for this remain unclear, it would be of interest to determine how the serum concentrations of IL-36 family members are altered with age and whether these change under disease conditions. Arguably the most interesting question is to identify whether these observations underlie a true protective role for IL-36 in metabolic disease among patients and, if so, what are the specific mechanisms through which this is achieved? Although, IL-36β is elevated among IS patients, it appears that their peripheral mononuclear cells are relatively insensitive to IL-36β stimulation when compared to their IR counterparts, at least in terms of the induced secretion of IL-6 (Fig. s2A). IL-8 and IL-10 secretion were not significantly induced in response to IL-36β stimulation among any patient groups under these conditions (Fig. s2B, C). This altered responsiveness is perhaps reflective of a heightened peripheral inflammatory response associated with insulin resistance and indicates that IL-36 cytokines can drive proinflammatory responses in this setting as previously reported [6]. In an effort to further investigate altered IL-36 mediated responses between IS and IR patients, we also examined levels of *IL-1RL2, IL-1RAcP and MyD88* gene expression among PBMCs from HC and both insulin sensitive and insulin resistant CWO cohorts. However no differences in expression were observed and levels of IL-36 cytokines themselves were found not expressed at significant levels in patient PBMCs (Fig. s2D-F and data not shown).

Deeper mechanistic studies in clinical cohorts are warranted to address the relevance of these observations to the pathogenesis of obesity related metabolic dysfunction, in particular as children living with obesity transition towards adulthood and the increased risk of developing overt obesity related comorbidities.

### Supplementary information

Supplemental Methods

supplemental figure and table legends

Supplemental Figure 1

Supplemental Figure 2

Supplemental Table 1

## Data Availability

The data that support the findings of this study are available from the corresponding author upon reasonable request.
