# Supplementary material for: Serum IL-36β levels are associated with Insulin sensitivity in paediatric patients with obesity
Source: Int J Obes (Lond). 2024 Mar 11;48(7):1036–8. doi: 10.1038/s41366-024-01508-4 (PMC11216979; doi:10.1038/s41366-024-01508-4)
Supplement: Supplementary file 1 — Supplemental Methods [file 41366_2024_1508_MOESM1_ESM.docx]

**Materials and Methods:**

**Patient selection and exclusion criteria**

Following ethical approval (GEN/204/11) from the research ethics committee at Childrens Health Ireland (CHI)-Crumlin, Dublin, Ireland and informed written consent, blood samples were collected from a patient cohort of 112 children attending the endocrinology department at CHI-Crumlin. Exclusion Criteria were as follows: Patients with known underlying genetic conditions: (e.g. Trisomy 21, Turner Syndrome, Bardet-Biedl Syndrome, Prader-Willi Syndrome).Patients with known endocrine conditions: (e.g. Autoimmune Hypothyroidism, Idiopathic Growth Hormone Deficiency, Hypoparathyroidism).Patients with pre-existing autoimmune/inflammatory conditions: (e.g. Autoimmune Thyroidits, Type 1 Diabetes Mellitus, Juvenile Idiopathic Arthritis, Eczema, Inflammatory Bowel Disease).Patients with diagnosed immunodeficiencies: (e.g. Bruton’s Aggamaglobulinemia, IgG defects). Patients who had received recent immunosuppressive treatment: (e.g. oral/inhaled/intravenous steroids or other immunomodulator therapy including Tacrolimus in the preceding 2 weeks).Patients with evidence of current active infection: (e.g. current intercurrent infection or use of antibiotics in the preceding 3 weeks).

**Clinical measurements**

For each child enrolled in the study, a series of clinical data were recorded, including age, sex, height, weight, medical history, current medications and blood pressure. Each subject also underwent a full physical examination. Peripheral blood samples were collected and glucose tolerance parameters were assayed using standardised methodologies. These parameters included fasting blood glucose, fasting insulin and haemoglobin A1c.

**Subclassification of patients**

Patient weight and height were measured, and BMI (kilograms/metres^2^) calculated for each child. This index was subsequently converted into a BMI Z score (zBMI) that adjusts for both age and gender. The BMI Z score is recognized as the gold standard for objective clinical assessment of obesity in paediatric patients (Van den Broeck et al., 2009). On the basis of the BMI Z score, children can be categorised as underweight (zBMI <-2), healthy bodyweight (-2< zBMI <2), or obese (zBMI >2) (Barlow and Expert, 2007). Using this approach, our cohort of 112 children were subdivided into 51 healthy bodyweight children and 61 children with obesity (CWO), on the basis of their zBMI scores. To further subdivide our cohort of CWO with respect to severity of disease, we utilised a similar approach to that previously reported in adult patients with obesity with respect to patient response to insulin. This strategy uses the **HO**meostatic **M**odel **A**ssessment for **I**nsulin **R**esistance (HOMA-IR) model which predicts pancreatic-cell function and insulin sensitivity for a given combination of fasting levels of plasma glucose and insulin (Muniyappa et al., 2008). HOMA-IR was calculated using the formula: HOMA-IR = Fasting Blood Glucose × Fasting Insulin/22.5. Using this model, all children with obesity enrolled in the study were sub-classified as being either insulin sensitive (HOMA-IR <3) or insulin resistant (HOMA-IR >3) respectively.

**PBMCs isolation and stimulation**

Peripheral Blood Mononuclear Cells (PBMC) were isolated by density centrifugation over ficoll, before cryopreservation in Cryostor CS10 (StemCell technologies). PBMCs were thawed and seeded in 24 well plates, at a density of 0.5 x 10^6^ cells per well, and immediately treated with IL-36β (100 ng/ml) for 48h. Supernatants were then collected and stored at -80 until used for cytokine determination.

**Measurement of cytokine levels by ELISA**

In order to determine cytokine levels in serum, peripheral blood samples were collected from each patient and allowed to clot. Serum samples were then obtained following centrifugation at 1000G for 10 minutes. Samples were then stored at -80ºC until used. To determine cytokine levels in PBMCs supernatants, these were collected after stimulation and stored at -80ºC until used. Levels of IL-18, IL-1β, IL-33, IL-36α, IL-36β, IL-36γ, and IL-36Ra in the serum and IL-6, IL-8 and IL-10 in the PBMCs supernatants were measured by ELISA using DuoSet Elisa Kits following the manufacturer’s instructions (R&D systems).

**RNA isolation**

Total RNA was obtained with Trizol reagent (TRI Reagent, Molecular Research Center, Inc., Cincinnati, OH, USA). Briefly, PBMCs were thawed and homogenized in Trizol. After centrifugation, nucleic acids and proteins were separated, and the aqueous phase containing the RNA was kept and precipitated in isopropanol. RNA yield and quality were determined and samples were stored at -80ºC until being used.

**Gene expression measurement by qPCR**

Reverse transcription was performed using High-Capacity cDNA kit (Applied Biosystems) and Real-time PCR was performed in duplicate using specific TaqMan Gene Expression Assays and TaqMan Fast Universal PCR Master Mix in a QuantStudio 3 System (Applied Biosystems). For normalisation, 18s ribosomal RNA was used, and relative gene expression levels were calculated by the ΔΔCt method.

**Statistical analysis**

For statistical analysis Prism 8 software (GraphPad) was used. All data was analysed for normality and homoscedasticity and the appropriate statistical test, unpaired Student *t* test or Mann-Whitney, was used as indicated. For correlation analysis, Spearman correlation was used. Statistical significance was determined as *p<0.05, **p<0.01, ***p<0.001 and ****p<0.0001.

Barlow, S. E. & Expert, C. 2007. Expert Committee Recommendations Regarding The Prevention, Assessment, And Treatment Of Child And Adolescent Overweight And Obesity: Summary Report. *Pediatrics,* 120 Suppl 4**,** S164-92.

Muniyappa, R., Lee, S., Chen, H. & Quon, M. J. 2008. Current Approaches For Assessing Insulin Sensitivity And Resistance In Vivo: Advantages, Limitations, And Appropriate Usage. *Am J Physiol Endocrinol Metab,* 294**,** E15-26.
