# Supplementary material for: Serum IL-36β levels are associated with Insulin sensitivity in paediatric patients with obesity
Source: Int J Obes (Lond). 2024 Mar 11;48(7):1036–8. doi: 10.1038/s41366-024-01508-4 (PMC11216979; doi:10.1038/s41366-024-01508-4)
Supplement: Supplementary file 2 — supplemental figure and table legends [file 41366_2024_1508_MOESM2_ESM.docx]

**Figure s1: Serum levels of IL-36 family cytokines in healthy bodyweight controls and children with obesity.** Serum levels of **(A)** IL-36α **(B)** IL-36β **(C)** IL-36γ **(D)** IL-36Ra and **(E)** IL-18 in lean (n=51) compared to paediatric patients with obesity (n=61). (F-H) Relative serum levels of IL-36α, IL-36γ and IL-36Ra in children with obesity were sub-classified as either insulin sensitive (IS) or insulin resistant (IR) based on HOMA-IR score. Statistical analysis by two-tailed Mann-Whitney U test (* p ≤ 0.05).

**Figure s2: Altered IL-36 responsiveness of PBMCs from healthy bodyweight controls and both insulin-sensitive and insulin-resistant cohorts of children with obesity.** Secretion of **(A)** IL-6, **(B)** IL-8 and **(C)** IL-10 by PBMCs, cultured in the presence or absence of rIL-36β (100ng/ml) for 24hrs. Patients segregated on the basis of healthy bodyweight (n=17 for IL-6 and IL-8, n=10 for IL-10) and insulin sensitive children with obesity (n=11 for IL-6 and IL-8, n=8 for IL-10) and insulin resistant children with obesity (n=14, n=9 for IL-10). **(D-F)** Ex vivo gene expression analysis of *IL1RAcP, IL1RL2* (*IL36R)* and *MyD88* from PBMC derived from healthy bodyweight controls (n=9), insulin sensitive children with obesity (n=6) and insulin resistant children with obesity (n=9). Statistical analysis by two-tailed Mann-Whitney U test (* p ≤ 0.05, ** p ≤ 0.01).

**Table s1: Patient Cohort information.** Clinical data shown as mean with range of values recorded in parentheses. Clinical parameters were compared between healthy bodyweight controls and insulin sensitive or insulin resistant children with obesity, by Mann-Whitney U test (* p ≤ 0.05, ** p ≤ 0.01, *** p ≤ 0.001).
