# Supplementary figures and images for: Serum IL-36β levels are associated with Insulin sensitivity in paediatric patients with obesity
Source: Int J Obes (Lond). 2024 Mar 11;48(7):1036–8. doi: 10.1038/s41366-024-01508-4 (PMC11216979; doi:10.1038/s41366-024-01508-4)

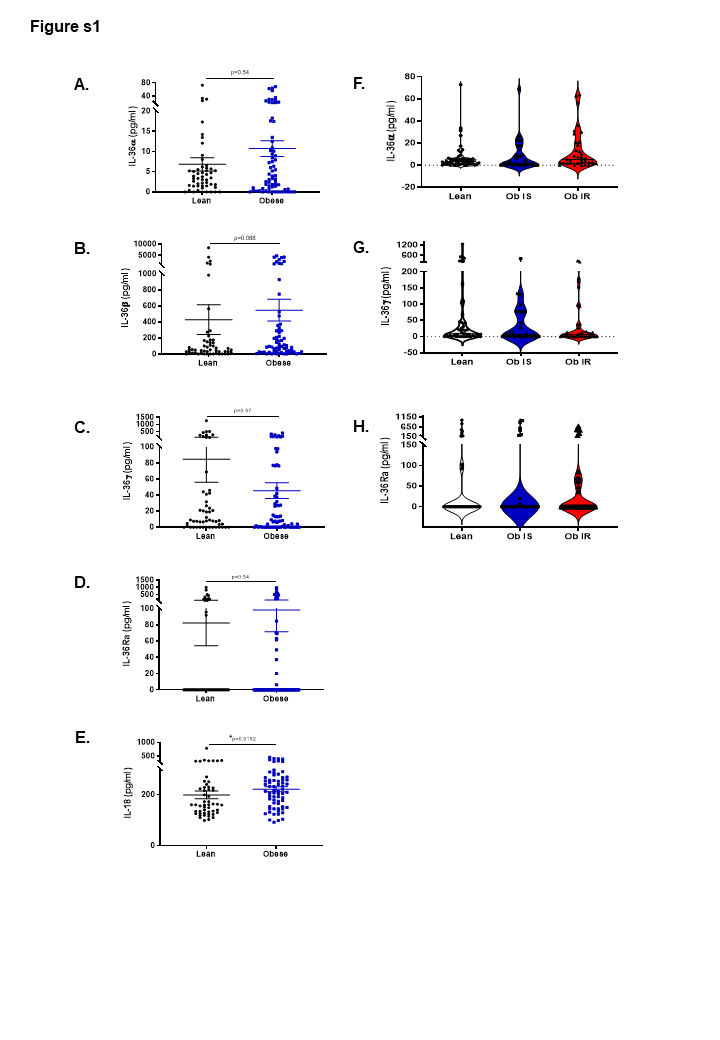

Supplement: Supplementary file 3 — Supplemental Figure 1 [file 41366_2024_1508_MOESM3_ESM.tif]

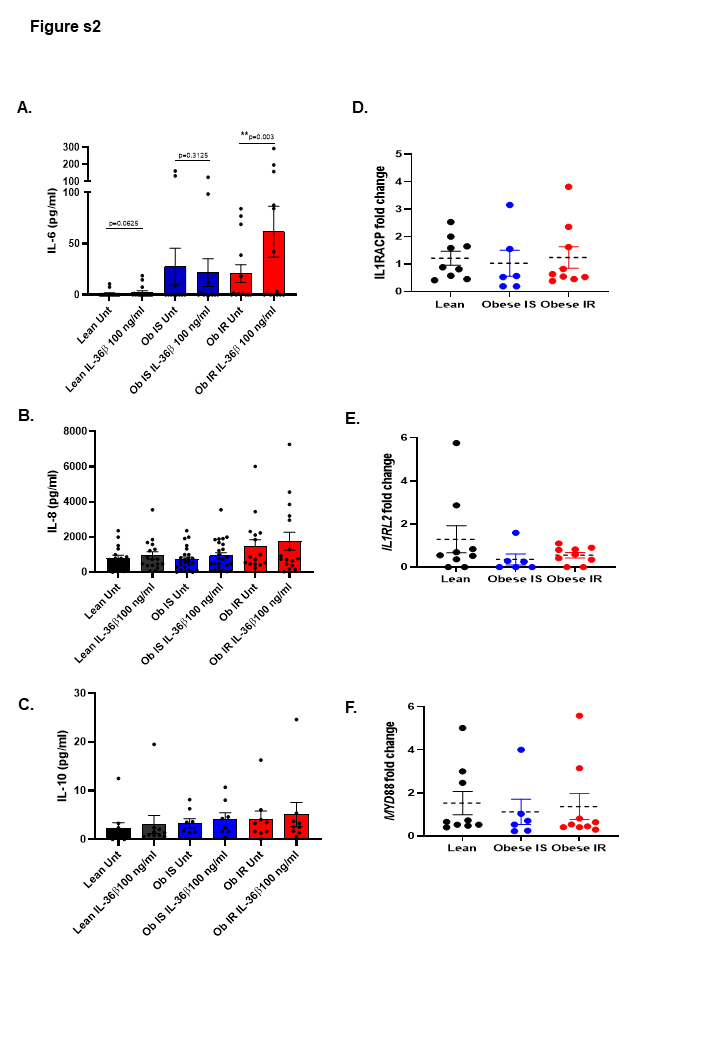

Supplement: Supplementary file 4 — Supplemental Figure 2 [file 41366_2024_1508_MOESM4_ESM.tif]

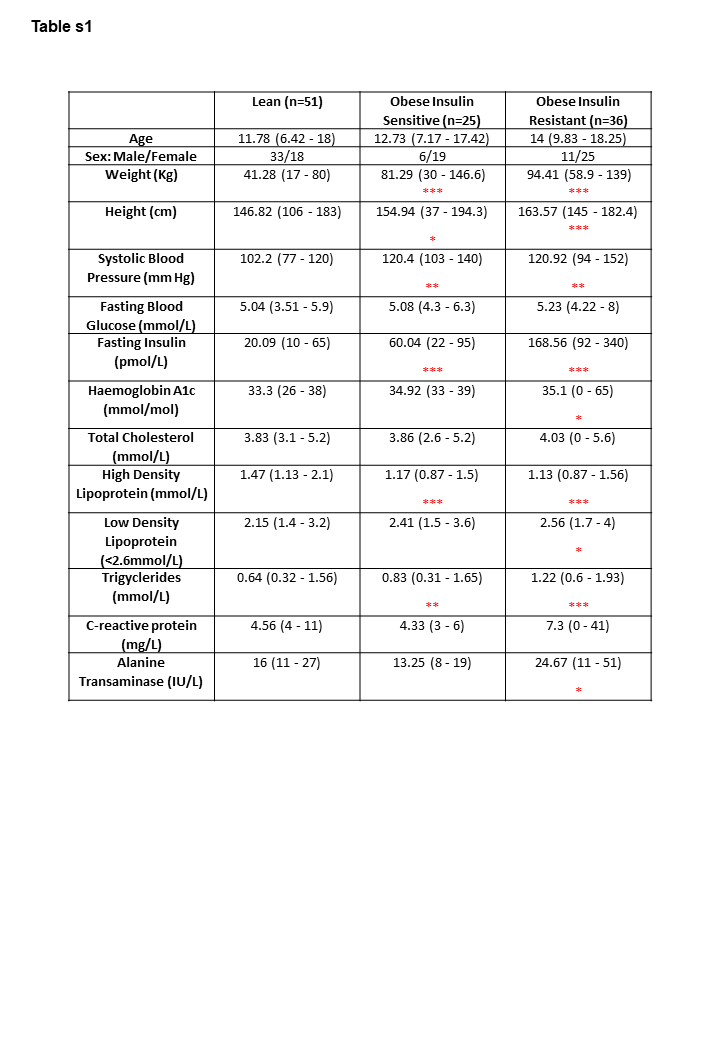

Supplement: Supplementary file 5 — Supplemental Table 1 [file 41366_2024_1508_MOESM5_ESM.tif]
